# Quantitative analysis of conjunctival microvasculature imaged using optical coherence tomography angiography

**DOI:** 10.1186/s40662-019-0130-9

**Published:** 2019-02-02

**Authors:** Zhiping Liu, Hua Wang, Hong Jiang, Giovana Rosa Gameiro, Jianhua Wang

**Affiliations:** 1 Ophthalmic Center, the Second Affiliated Hospital of Guangzhou Medical University, Guangzhou, Guangdong China; 2 Department of Ophthalmology, Bascom Palmer Eye Institute, University of Miami Miller School of Medicine, 1638 NW 10th Avenue, McKnight Building - Room 202A, Miami, FL 33136 USA; 3 Department of Ophthalmology, Xiangya Hospital, Central South University, Changsha, Hunan China

**Keywords:** Conjunctival microvasculature, Optical coherence tomography angiography (OCTA), Functional slit lamp bio-microscopy (FSLB), Anterior segment

## Abstract

**Background:**

The goal was to quantitatively analyze the bulbar conjunctival microvascular density using optical coherence tomography angiography (OCTA) and compare it to the vessel density using functional slit-lamp biomicroscopy (FSLB).

**Methods:**

Temporal bulbar conjunctiva of 20 eyes (10 healthy subjects) was imaged using both OCTA and FSLB. Image processing was performed including equalization, de-noising, thresholding, and skeletonization. The vessel density was measured by fractal analysis (box counting, Dbox) and pixel counting (%).

**Results:**

Vessel density (Dbox) of the bulbar conjunctiva obtained using OCTA was 1.28 ± 0.01 Dbox, which was significantly lower than the result (1.32 ± 0.01 Dbox, *P* < 0.001) obtained using FSLB. Furthermore, the vessel density (%) obtained using OCTA was 3.31 ± 0.12%, which was also significantly lower than the result (3.69 ± 0.16%, *P* < 0.001) obtained using FSLB. No significant correlations (r ranged from 0.21 to 0.32, *P* > 0.05) between both instruments were found in both vessel density methods (Dbox and percentage). However, in each of the devices, vessel density in Dbox was significantly correlated with the vessel density in percentage (*r* = 1.0 for FSLB and *r* = 0.98 for OCTA, both *P* < 0.001).

**Conclusion:**

This study demonstrated that the vessel density of the bulbar conjunctiva obtained using OCTA can be quantified, and the results were not compatible with that obtained using slit-lamp biomicroscopy photography.

## Background

The vessels of the bulbar conjunctiva are branches from the ophthalmic artery, which could be affected by infection, allergy, dry eye and some systemic diseases, such as diabetes and hypertension [1–4]. The conjunctival microvascular network can be easily visualized and is feasible for in vivo imaging to investigate the physiological or pathological changes in ocular [4], systemic [1], and central nervous system (CNS) vascular diseases [5].

Optical coherence tomography angiography (OCTA) is a non-invasive imaging modality for imaging the vessels with depth information. It is commonly used for imaging the retinal vasculature [6–9]. Recently, OCTA has been employed in imaging the anterior segment of the eye for aiding the diagnosis of vascular lesions of the conjunctiva, cornea, and iris [10–13]. While the analysis of vessel density in the retina was reported in many previous studies [14–16], the vessel density of the anterior segment was reported in several publications [10, 17–21]. Akagi et al. reported the vessel quantification using pixel counting and fractal analysis for analyzing vessel density in healthy subjects [17]. The underdevelopment of the anterior segment vessel imaging and its analysis may be due to the difficulty in image acquisition (attributable to having no standard protocol) and image analysis of these OCTA images with a relatively high signal to noise ratio [17].

Compared to OCTA for acquiring the angiogram of the conjunctiva with the scanning method, slit-lamp biomicroscopy can also be used to acquire the vessel information in the conjunctiva through photography [4, 22, 23]. The slit-lamp photography method such as functional slit-lamp bio-microscopy (FSLB) was used to image the conjunctival microvasculature in healthy subjects, contact lens wearers, and patients with dry eyes [22, 24]. Although the FSLB only acquires the vessels in the depth where the illumination light penetrates, it is not clear whether the vessel densities obtained using both OCTA and FSLB are compatible. The goal was to quantitatively analyze the bulbar conjunctival microvascular density imaged using OCTA and compare the OCTA vessel density to the one imaged using FSLB.

## Materials and methods

This was a prospective study; the study protocol was approved by the institutional review board of the University of Miami. Ten self-reported healthy volunteers were enrolled in this study. All subjects were treated according to the tenets of the Declaration of Helsinki. After risks and benefits of participation were explained to the participants, written informed consent was obtained from each of the subjects. All subjects underwent comprehensive ophthalmic examinations including best corrected visual acuity and manifest refraction. All subjects were self-reported healthy subjects without any ocular pathology (such as pterygium, pingueculum, ocular surface tumor, keratitis and dry eye), previous ocular surgeries and systemic diseases (such as stroke, hypertension and diabetics). These participants had an average age of 33.3 ± 10.1 years (range: 19–53 years; Table 1).

**Table 1:** Demographic information

	Mean	±	SD		Range	
No. of subjects		10				
Female/Male		8F 2M				
Age (years)	33.3	±	10.1	19	~	53
SBP (mmHg)	108.9	±	9.0	92	~	119
DBP (mmHg)	73.2	±	7.6	62	~	89
HR (beats/min)	70.6	±	8.8	60	~	86

*SBP=* systolic blood pressures, *DBP=* diastolic blood pressures, *HR=* heart rate

**Fig. 1:**
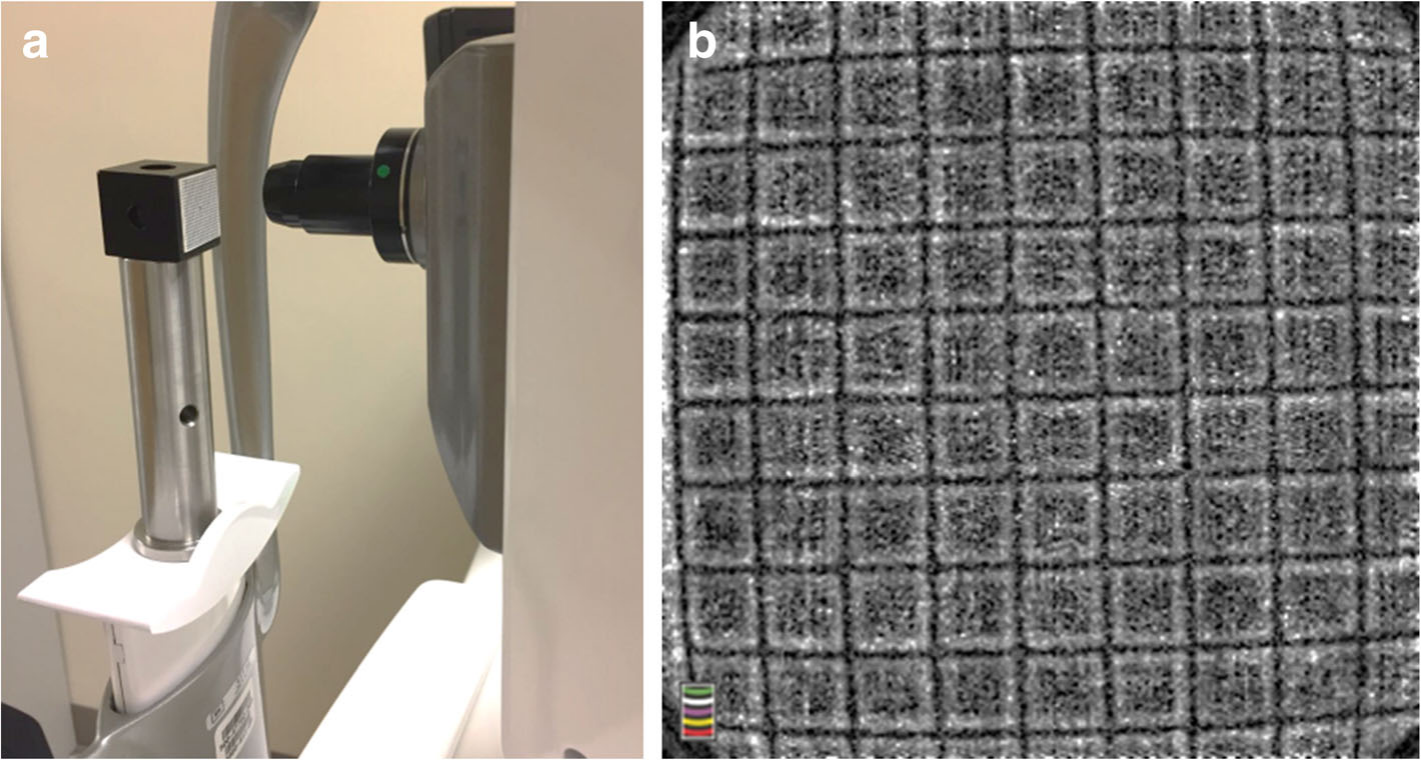
Calibration of the field of view (FOV) using OCTA. **a** A target with a printed grid (interval of 1 mm) was placed in front of the instrument with an anterior segment imaging lens, and a 6 × 6 mm^2^ raster scan was used to scan the target. The system parameters were set as: Z Motor = 15; Focus = − 15 and P Motor = 20. **b** The FOV of the OCTA with the 6 × 6 mm^2^ scan was calibrated as 8.775 × 8.775 mm^2^

To determine the field of view (FOV) of OCTA for imaging the anterior segment, a target with a printed grid (interval of 1 mm) was placed in front of the instrument with an anterior segment imaging lens (adapter) provided by the manufacturer, and a 6 × 6 mm^2^ raster scan was used to scan the target. The FOV of the OCTA with the 6 × 6 mm^2^ scan protocol was calibrated as 8.775 × 8.775 mm^2^ (Fig. 1).

The AngioVue OCTA system (Optovue, Fremont, California, USA) was used to image the conjunctiva. The AngioVue OCTA system has been described previously [10]. Briefly, the system is a spectral domain OCTA system with a scan speed of 70,000 A-scans per second, and the axial resolution is 5 μm. The transverse resolution is dependent on the scan area and distribution of A-scans. The width of the OCT scan beam is 22 μm with a light source centered on 840 nm. A typical OCTA scan contains a raster scan with 304 (A-scan) × 304 (B-scan). It takes about 3–4 s to obtain one raster volumetric scan, and two scans are needed to create an angiogram using proprietary angiography algorithms and motion correction technique (MCT) [25]. The exported *en face* OCTA vessel image is dependent on the segmented depth (boundary).

In this study, the Angio Retina 6 × 6 mm^2^ scan protocol was used with the OCTA system, and an anterior segment scan lens was mounted (Fig. 1). The subject was asked to look at an external fixation target at the nasal side so that the temporal side of the study eye was exposed to the scanning probe. Normal blinking was allowed during imaging.

The FSLB and imaging procedure have been previously described [6, 16]. Briefly, the FSLB is a standard slit-lamp attached to a complementary metal-oxide-semiconductor (CMS) based digital camera with an imaging sensor size of 22.3 × 14.9 mm^2^, which acquires a maximum resolution of 5184 × 3456 pixels. The pixel dimension on the camera sensor is 4.3 × 4.3 μm^2^. To photograph the temporal conjunctiva, a built-in green filter and 15x magnifications were used. The International Standards Organization (ISO) sensitivity was set to be 2500, and exposure time was set to be 1/15 s. The calibrated FOV of this setting is 15.74 × 10.50 mm^2^.

During FSLB imaging, the subject was looking at a target at the nasal position while the temporal conjunctiva was imaged. By observing the live-view on a high-definition monitor, the middle of the temporal conjunctiva was focused before a series of still images were captured [15, 21]. To segment conjunctival vessels, the raw images were resized from 5184 × 3456 pixels to 1024 × 683 pixels. Custom software (Mathworks, Inc., Natick, MA) was then used to extract the vessels using a series of image processing procedures as described in previous publications [4, 24]. Of note, the raw image of the conjunctiva was resized and converted to the grayscale. After equalization using adaptive histogram equalization and morphological opening operation, the background noise and non-vessel structure were removed. The image in grayscale, which is similar to the image acquired using OCTA, was then resized to match the FOV of OCTA.

**Fig. 2:**
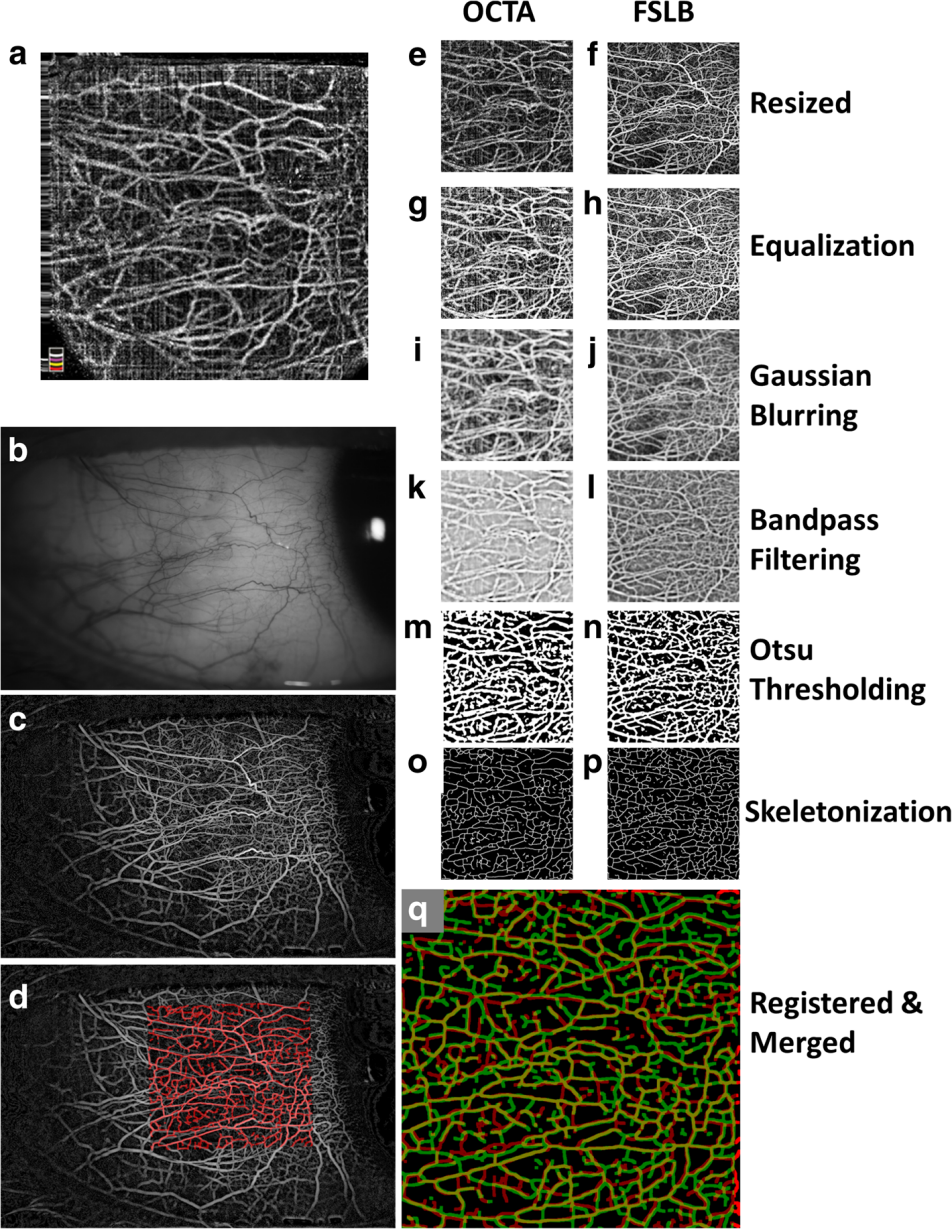
Image processing to extract the conjunctival microvascular network in OCTA and FSLB. **a** The raw OCTA image of the temporal bulbar conjunctiva (304 × 304 pixels, 8.775 × 8.775 mm^2^); **b** The raw FSLB image of the temporal bulbar conjunctiva (5184 × 3456 pixels, 15.74 × 10.50 mm^2^); **c** A custom software was used to segment the vessels from the raw image of FSLB to create the microvascular network by a series of image processing procedures. The image was resized to 1024 × 683 pixels in grayscale; **d** The segmented OCTA vessel image (skeletonized) was merged into the FSLB image; **e** The resized OCTA image with 768 × 768 pixels and the FOV was 6.581 × 6.581 mm^2^; **f** The resized FSLB image with 768 × 768 pixels and a FOV of 6.581 × 6.581 mm^2^; **g** Equalized OCTA image; **h** Equalized FSLB image; **i** OCTA image after Gaussian Blurring (Sigma = 4); **j** FSLB image after Gaussian Blurring (Sigma = 4); **k** OCTA image after bandpass filtering; **l** FSLB image after bandpass filtering; **m** Binary OCTA image after Otsu Thresholding; **n** Binary FSLB image after Otsu Thresholding; **o** Skeletonized OCTA image after Otsu Thresholding; **p** Skeletonized FSLB image after Otsu Thresholding; **q** Merged skeletonized images obtained using OCTA and FSLB. Yellow: vessels extracted from both devices; red: vessels only extracted from OCTA, and green: vessels only extracted from FSLB

The raw images of OCTA were exported as the *en face* OCTA images with the full thickness of the conjunctiva and resized from 304 × 304 pixels to 1024 × 1024 pixels (8.775 × 8.775 mm^2^) and saved as BMP images. The resized images were cropped to 768 × 768 pixels, and the FOV was set as 6.581 × 6.581 mm^2^ (Fig. 2). To make sure the conjunctival area had the same size and was on a similar location for both OCTA and FSLB, the resized OCTA image was registered into the raw FSLB image for aiding the cropping of the FSLB image. The cropped FSLB was then resized to 768 × 768 pixels with the FOVs of the same size of OCTA (Fig. 2).

The cropped and resized images with the same image size and FOV obtained using OCTA and FSLB were processed in Photoshop (Adobe, San Jose, CA, USA) and ImageJ (NIH Bethesda, Maryland, USA) (Fig. 2). Equalization and conversion to a grayscale mode were performed in Photoshop. After that, these images were processed in ImageJ with a series of filtering and thresholding including Gaussian blurring (Sigma = 4), bandpass filtering (filter large structures down to 40 pixels, and small structures down to 3 pixels, no saturation), and Otsu’s Thresholding. Gaussian blurring is an image process that blurs an image using a Gaussian function that is commonly used to reduce image noise [26]. The Otsu’s Thresholding is a method used to perform thresholding on the contents and convert the image to a binary image [17]. The algorithm of the Otsu’s Thresholding assumes that the image contains two classes of pixels following bi-modal histogram (foreground pixels and background pixels), and calculates the optimum threshold separating the two classes [27]. The binary image was skeletonized for calculating vessel density and fractal analysis. Vessel density was defined as the percentage of pixels with vessels in the skeletonized image over the entire image. Fractal analysis (box counting, Dbox, representing vessel density) was performed using Benoit Software (TruSoft Benoit Pro 2.0; TruSoft Inc., St Petersburg, Florida, USA). The setting of the fractal analysis was 104 of side-length of the largest box and 15 of increment of box sizes.

## Statistical analysis

All values are presented as mean ± standard deviation. All analyses were performed using IBM SPSS Statistics 25 (IBM Corp., Armonk, NY, USA). Differences in the two vessel density measurements (Dbox and %) between OCTA and FSLB were analyzed using repeated measures analysis of variance (Re-ANOVA), and post hoc tests were used to test pair-wise differences. Pearson’s regression was used to determine the relationships among parameters. The Bland-Altman plot was used to determine the 95% limit of agreement, which was calculated as 1.96 × the standard deviation of the difference between the measurements using OCTA and FSLB. *P* values less than 0.05 were considered statistically significant.

## Results

In our present study, a cohort of 20 eyes provided a statistical power of 100% in differentiating the vessel densities expressed in Dbox or percentage between these two devices. The calculation was based on the measurements using both devices of the cohort in the present study. Although the vessels on the conjunctiva acquired using both imaging devices were similar, it appeared that the vessels acquired using OCTA were lesser, compared to the ones acquired using FSLB (Fig. 2). In the merged image, it appeared that there were more vessels extracted from the FSLB image than vessels extracted from the OCTA image, although the vessels extracted from both devices were predominant.

**Fig. 3:**
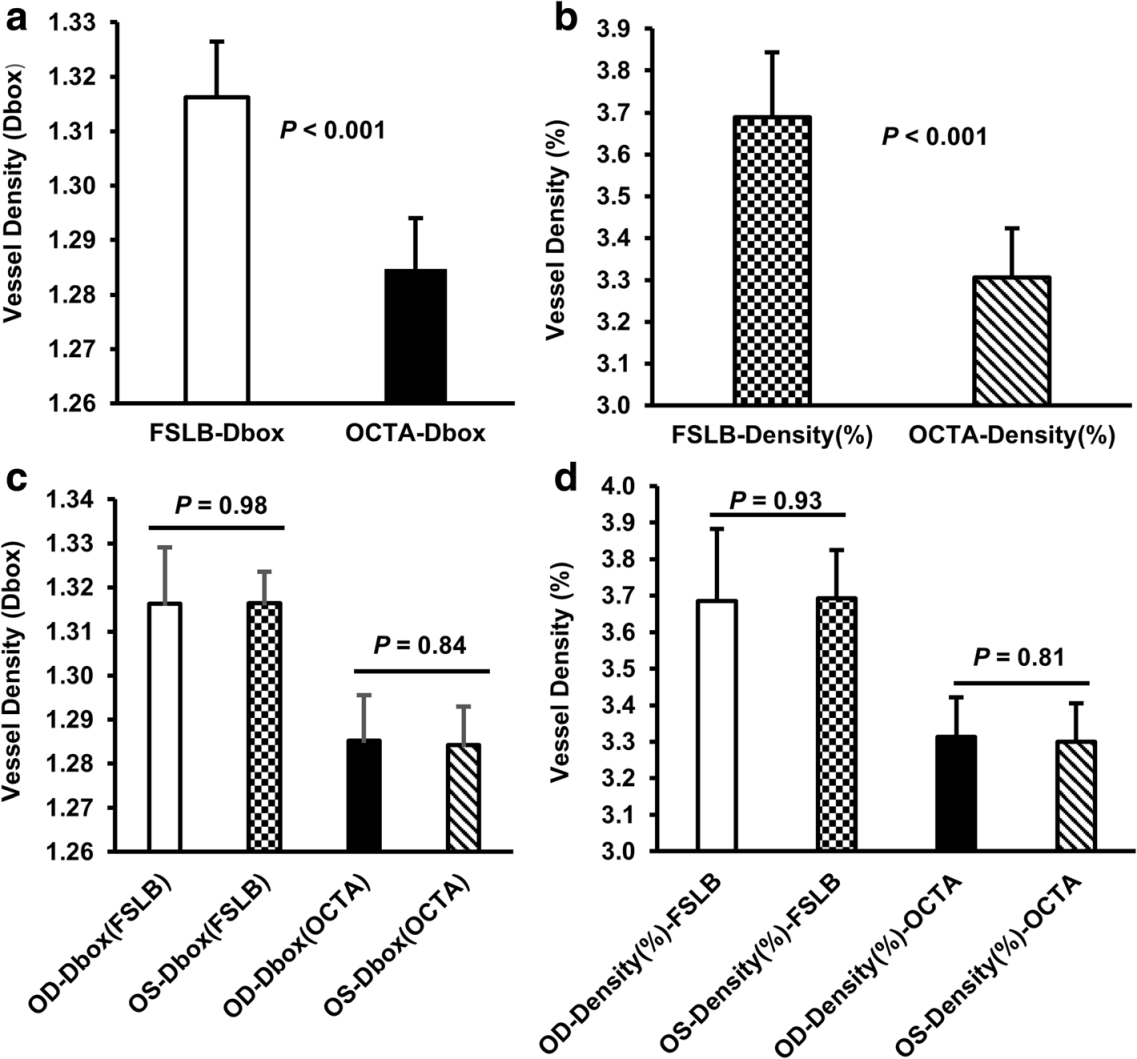
Vessel densities (Dbox and %) in the conjunctival microvascular network measured using OCTA and FSLB. **a** Significant differences were found in vessel density (Dbox) between FSLB and OCTA (*P* < 0.001). **b** There also were significant differences in vessel density (%) between FSLB and OCTA (*P* < 0.001). **c** Vessel densities (Dbox) acquired using both FSLB and OCTA were not different between the right and left eyes (*P* > 0.05), and (**d**) Vessel densities (%) acquired using both FSLB and OCTA were not different between the right and left eyes (*P* > 0.05)

Vessel density (Dbox) of the conjunctival microvasculature obtained using OCTA was 1.28 ± 0.01 Dbox, which was significantly lower than the result (1.32 ± 0.01 Dbox, *P* < 0.001, Fig. 3) obtained using FSLB. Furthermore, vessel density (%) using pixel counting obtained using OCTA was 3.31 ± 0.12%, which was significantly lower than the vessel density (3.69 ± 0.16%, *P* < 0.001) obtained using FSLB.

**Fig. 4:**
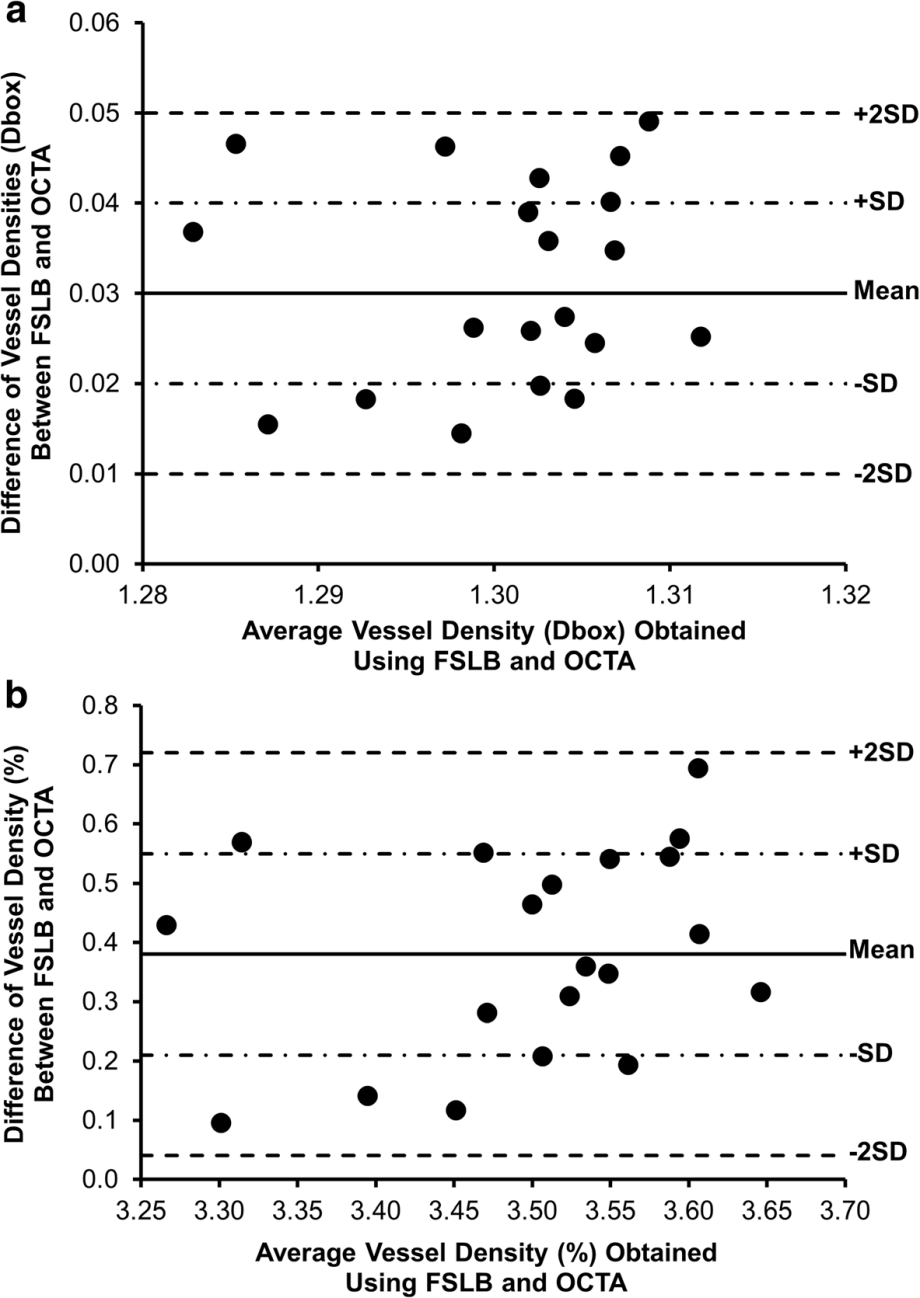
Bland-Altman Plot of vessel density (Dbox and percentage) acquired using OCTA and FSLB. **a** Bland-Altman plot shows the limit of agreement of the vessel density (Dbox). **b** Bland-Altman plot shows the limit of agreement of the vessel density (%). Note that the solid and dashed lines indicate the mean difference and 95% limit of agreement. Systematic differences between devices are evident

There were no significant differences in vessel densities measured with both devices between the right and left eyes (*P* > 0.05, Fig. 3). Bland-Altman plots of the difference against the mean vessel densities (Dbox and percentage) showed the limit of agreement of these measurements although systematic differences existed (Fig. 4).

**Fig. 5:**
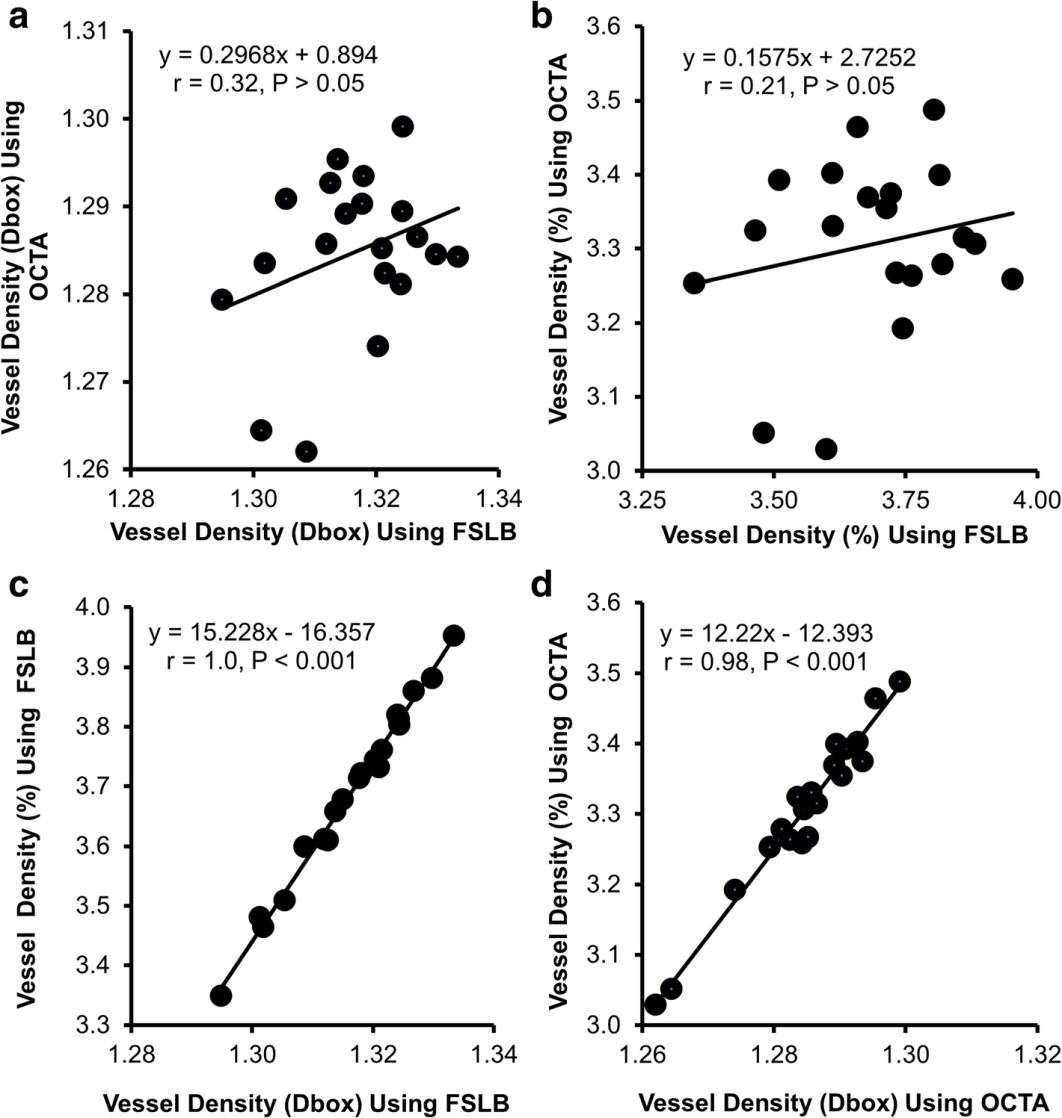
Relationships among vessel densities acquired using OCTA and FSLB. No significant correlations were found in vessel densities (Dbox and percentage) between OCTA and FSLB (*P* > 0.05, **a** and **b**). However, with each of the devices, vessel density in Dbox was significantly correlated with the vessel density in percentage (r = 1.0 for FSLB and r = 0.98 for OCTA, both *P* < 0.001, **c** and **d**)

No significant correlations were found in vessel densities (Dbox and percentage) between OCTA and FSLB (*P* > 0.05, Fig. 5). However, with each of the devices, vessel density in Dbox was significantly correlated with the vessel density in percentage (*r* = 1.0 for FSLB and *r* = 0.98 for OCTA, both *P* < 0.001, Fig. 5).

## Discussion

The microvasculature on the bulbar conjunctiva is a very dense network with a high level of complexity [17]. There is an anastomotic microvascular network of capillaries, arterioles, and venules around the limbus, which could be readily imaged. It makes the bulbar conjunctiva an ideal tissue for noninvasive imaging of pathologic conditions and evaluations of therapies [24]. OCTA has been used to image vessels in the anterior segment in healthy subjects and vascular diseases (Table 2) [28–30]. OCTA can detect vascular lesions in anterior segment tissues and display an angiogram of their layers. Since OCTA provides depth information of the scanned tissue, pathological changes of blood vessels in different layers can be segmented and visualized. In addition to visualization of the depth encoded angiogram, quantification of vessel density may further boost OCTA in widespread use in research and routine clinics.

**Table 2:** Summary of OCTA study of the anterior segment of the eye

Reference	Eyes	Age (mean ± SD, years)	OCTA Company	Target	Condition	Quantification
Present Study	20	33.3 ± 10.1	Optovue, Fremont, CA, USA	Conj	NE	VD
Akagi et al., 2018 [17]	10	28.5 ± 7.1	Carl Zeiss Meditec, Dublin, CA, USA	Conj / ISV	NE	VD/VLD/VDI/FD
Ang, et al., 2016 [18]	8	45 ± 0.5	Optovue, Fremont, CA, USA	KNV	KNV	AOV
Allegrini, et al., 2016 [38]	28	/	Optovue, Fremont, CA, USA	Iris	NE	/
Roberts, et al., 2017 [39]	50	50 ± 18	Optovue, Fremont, CA, USA	Iris	NE/ INV	/
Ang, et al., 2018 [19]	10	23–73	AOptovue, Fremont, CA, USA, Angioscan, Nidek Co Ltd., Japan	KNV	KNV	VD
Zett, et al., 2018 [30]	20	/	Optovue, Fremont, CA, USA	Iris	IP	/
Williams, et al., 2018 [40]	22	58–82	Optovue, Fremont, CA, USA	Iris	IM	/
Skalet, et al., 2017 [20]	17	40.1 ± 10.9/ 66.4 ± 18.0	Optovue, Fremont, CA, USA	Iris	NE/IM/BIL	VD
Ang, et al., 2015 [21]	30	49.27 ± 17.23	Optovue, Fremont, CA, USA	KNV	KNV	AOV
Ang, et al., 2015 [10]	40	25.3 ± 7.8	Optovue, Fremont, CA, USA	ASV	NE/CNV	VM

*Conj=* conjunctiva, *ISV=* intrascleral vasculatures, *KNV=* corneal neovascularization, *ASV=* anterior segment vasculature, *INV=* iris neovascularization, *NE=* normal eyes, *IP=* iris pigmentation, *IM=* iris microhemangiomatosis, *BIL=* benign iris lesion, *VD=* vessel density, *VLD=* vessel length density, *VDI=* vessel diameter index, *FD=* fractal dimension, *AOV=* area of vascularization, *VM=* vasculature measurements

As OCTA is a relatively new modality, quantification of OCTA for both the posterior and anterior segments is underdeveloped, and currently there is no gold standard for vessel quantification. To quantify the vessels acquired using OCTA, fractal analysis [16, 31] and pixel counting [32, 33] are used to analyze retinal vessel density, sometimes called capillary perfusion density [34]. Fractal analysis is a mathematical method used to analyze fractal characteristics of data containing objects and patterns, which are commonly analyzed using box counting method [24]. Here, fractal dimension (Dbox) represents the vessel density. In contrast, pixel analysis is counting the pixel containing the objects (i.e., vessels) over a certain area, which is normally expressed as percentage [17]. Similar approaches have also been used in quantifying vessel density of the anterior segment of the eye [11, 17] (Table 2). Robust image processing was demonstrated by Akagi et al. who used swept light source OCTA to image the anterior segment in healthy subjects [17]. They used a series of image processing procedures including top-hat filter, bandpass filter, and Otsu’s thresholding. However, the authors acknowledged that significant noise still existed in OCTA images after image processing [17]. In this study, modified image processing procedures were used by applying equalization and Gaussian blurring for de-noising, in addition to the procedures described by Akagi et al. [17]. It appeared that most of the noise was removed while most vessels were extracted. Our results (Dbox) obtained using OCTA are very close to that reported by Akagi et al. [17]. As expected, the vessel density using skeletonized images was not in agreement with the vessel density using binary images without skeletonization. Skeletonization gives equal weight to each vessel regardless of its diameter, and this method is commonly used for calculating vessel density [15–17, 31, 34, 35].

Interestingly, the strong relations between the vessel densities using boxing accounting and pixel counting may indicate that the use of skeletonization of the vessel images is beneficial. This phenomenon also strongly supports the notion that fractal analysis using box counting represents vessel density. Therefore, vessel densities using boxing and pixel counting are interchangeable and can be converted. Either of these methods can be used for monitoring the vessel density.

As OCTA applies the scanning method to generate angiography with depth information, the scanning speed may limit its transverse resolution. Using the commonly used spectral domain OCTA devices like the AngioVue OCTA, the interval of each A-scan is about 25–30 μm, larger than the average diameter of the conjunctival vessels [17, 24, 36]. This calculation may explain the reason why the OCTA-derived vessel density was lesser than FSLB-derived vessel density, as demonstrated in the present study. Although FSLB does not provide depth information, the pixel interval is 4.3 μm in the camera sensor and resized to 20 μm after resizing. Also, the capture speed of the FSLB is 1/15 s compared to 3–4 s of OCTA scanning for one volumetric dataset. The slow capture speed of OCTA may also contribute to the high noise level of the OCTA *en face* images. Based on the comparison of the vessel densities acquired from different modalities, it appears that the results from OCTA are not comparable to the results acquired using FSLB, although similar image processing and analysis could be separately applied to both images. Since these vessel density measurements are not related and not comparable, we do not recommend converting the measurements between these two different modalities.

It is worth noting that the most important advantage of OCTA is the depth information in angiogram, which can be used to segment the vessels in different layers such as the conjunctiva and sclera [17]. Akagi et al. used OCTA equipped with the swept light source at the center wavelength of 1 μm to assess conjunctival and intrascleral vasculature in normal eyes, which opens a new application of OCTA in imaging the anterior segment of the eye [17]. We examined our OCTA images and found that the vasculature of the sclera (in the deep layers below 300 μm from the conjunctival epithelium) was visualized, although the deep image was dark due to the use of a light source with a relatively short wavelength (840 nm) in the AngioVue OCTA device [21]. In contrast, FSLB is based on photography which cannot provide depth information [24, 37], resulting in incomparability between these two devices. This may partially explain why the vessel densities obtained from both devices were not comparable.

Our present study provided a simple solution using readily available software programs for image processing to quantitatively analyze OCTA vessel density and the information about the comparability to the photography of the conjunctival vessels. There are some limitations. First, there are no diseased eyes in the present study, which makes it impossible to compare the discriminatory powers of the OCTA vessel analysis of the anterior segment compared to FSLB. Second, the sample size may be small although we demonstrated the differences in vessel densities acquired using different image modalities. Further studies with a large sample size are needed. Third, conjunctival vasculatures are different in different quadrant and depth [17] but we only used the angiogram from the full thickness of the conjunctiva in an attempt to compare OCTA and FSLB. Previous studies using FSLB commonly imaged the temporal side of the conjunctiva [24, 37]. Therefore, we selected the temporal side of the conjunctiva in the present study. Further studies will need to analyze other locations and vessel layers in normal and diseased eyes. The image processing approach described in this study will need to be further tested in the angiogram from different layers of the conjunctiva similar to the analysis done by Akagi et al. [17]. Fourth, we did not test the repeatability of our image processing method in the analysis of conjunctival vessels. The inter-eye variability may serve as a rough reference of the repeatability. However, further studies are also needed to test inter- and intra-visit variables of the vessel density measurements.

## Conclusions

This study demonstrated that vessel density of the bulbar conjunctiva obtained using OCTA can be quantified, and the results were not compatible with that obtained using slit-lamp biomicroscopy photography.
